# Emphysematous Pyelonephritis From a Perinephric Hematoma Complicated by Fournier’s Gangrene: A Case Report

**DOI:** 10.7759/cureus.21612

**Published:** 2022-01-25

**Authors:** Benjamin A Fink, Young Son, Brian Thomas, Thomas J Mueller, Douglas S Berkman

**Affiliations:** 1 Urology, Rowan University, School of Osteopathic Medicine, Stratford, USA; 2 Urology, Jefferson Stratford Hospital, Stratford, USA; 3 Urology, New Jersey Urology, Voorhees, USA

**Keywords:** scrotum, radical orchiectomy, retroperitoneal hematoma, emphysematous pyelonephritis, fournier gangrene

## Abstract

Fournier’s gangrene and emphysematous pyelonephritis are rare necrotizing infections of the genitourinary system. Many cases of this rapidly progressive infection occur from abscesses and urinary tract infections; however, Fournier’s gangrene secondary to emphysematous pyelonephritis is seldom discussed in the literature. Emphysematous pyelonephritis is defined as a gas-forming, necrotizing infection of the renal parenchyma or its surrounding tissue. Emphysematous pyelonephritis has been observed in high-risk individuals, including those with poor glycemic control and urinary tract obstruction. We present a 61-year-old male with emphysematous pyelonephritis arising from a perinephric hematoma with tracking of the infection to the scrotum, resulting in Fournier’s gangrene. The perinephric hematoma most likely developed from increased intrarenal hydrostatic pressure during nephroureteral stent placement. Broad-spectrum antibiotic therapy and surgical debridement of the retroperitoneum, groin, and scrotum were performed ultimately requiring left orchiectomy. We conclude that an existing hematoma can precipitate emphysematous pyelonephritis with tracking from the retroperitoneum to scrotum, causing Fournier’s gangrene. High-risk patients with perinephric hematomas can be susceptible to this pathologic transformation.

## Introduction

Fournier’s gangrene is an uncommon, frequently fatal, polymicrobial necrotizing fasciitis of the scrotum, penis, perineum, or perianal region. Risk factors for this infection include male gender, diabetes, human immunodeficiency virus (HIV), and other immunocompromised states [1]. Diabetes mellitus, in particular, is a primary risk factor due to increased incidence of urinary tract infection, defective phagocytosis, and vasculitis [2]. End-stage renal disease, malnutrition, and high body mass index also serve as risk factors due to decreased patient immunity and therefore increased susceptibility to infection [3]. Several mechanisms have been suggested to underlie the development and progression of Fournier’s gangrene. Intestinal perforation, surgical instrumentation, and perianal abscesses are common causes discussed in the literature [4,5]. 

Emphysematous pyelonephritis can be another source of Fournier’s gangrene [6]. Emphysematous pyelonephritis is a necrotizing infection of the renal parenchyma or its surrounding tissue with gas production [7]. This renal infection typically develops with poor glycemic control or urinary tract obstruction [8]. Emphysematous pyelonephritis is commonly treated with medical management including broad-spectrum antibiotics, percutaneous drainage, relief of obstruction, or emergent nephrectomy [9]. Very few cases have identified the development of emphysematous pyelonephritis from an existing hematoma [10]. Furthermore, a limited number of cases have outlined a distinct pathway of emphysematous pyelonephritis extension from the retroperitoneum to the scrotum ultimately leading to Fournier’s gangrene. The etiology of retroperitoneal gangrene tracking to the scrotum is ambiguous and difficult to identify in literature [11]. 

Common bacteria isolated from Fournier’s gangrene patients usually represent normal flora of the urogenital and gastrointestinal tracts, including gram-negative rods (*Escherichia coli*, *Klebsiella *spp., *Proteus* spp.), gram-positive cocci (*Staphylococcus*, *Streptococcus*, *Enterococcus*), and obligate anaerobic bacteria (*Clostridium* spp., *Fusobacterium* spp.) [12].

The immediate treatment recommended for Fournier’s gangrene includes hemodynamic support with fluids, broad-spectrum antibiotics, and most importantly, surgical debridement. Mortality from Fournier’s gangrene has been estimated at almost 20% even with aggressive treatment protocols [13]. Traditionally, antibiotic therapy includes carbapenem or piperacillin-tazobactam, vancomycin, and clindamycin [12]. Multiple surgical debridements are often required with early surgical treatment proven to decrease mortality rates. Orchiectomy is rarely required in cases of Fournier's gangrene due to the distinct infectious spread fascial layer [14]. There has been no reported literature of gas-forming organisms developing from an existing retroperitoneal hematoma with an extension of infection to the scrotum causing Fournier’s gangrene.

## Case presentation

**Figure 1:**
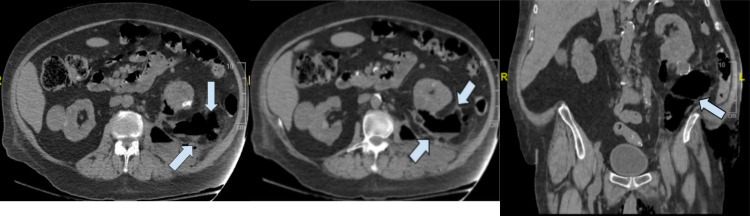
CT cross sectional images The left and middle panels are transverse sections and the right panel is a coronal section showing air forming infection starting in the left retroperitoneum extending to the left scrotum (arrows).

A 61-year-old male with a history of end-stage kidney disease, diabetes mellitus, gross hematuria, status post ureteroscopy and ureteral stent placement seven months ago complicated by perinephric hematoma, presented to the emergency department (ED) with left-sided scrotal pain and swelling radiating to the left groin and back. The patient reported symptom onset 12 hours prior to presentation. The physical evaluation demonstrated crepitus of the left scrotum and left flank pain on palpation. On arrival, the patient’s hemoglobin was 9.1 grams per deciliter (g/dL), serum creatinine of 3.2 milligrams (mg)/deciliter (dL), white blood cell (WBC) count of 8.7 x 10^3^ per uL (microliter) and a blood lactate of 3.1 mg/dL. His medication history included alprazolam, amiodarone, methoxy peg-epoetin beta, and nebivolol. The patient was started on piperacillin-tazobactam, vancomycin, and clindamycin in the ED. Computed tomography (CT) without contrast revealed complex abscesses within the left retroperitoneal space adjacent to the inferior aspect of the left kidney with extensive air along the soft tissue planes tracking to the perineum. In addition, a 10.0 x 5.0 centimeter (cm) hyperdense collection with air involving the left iliopsoas muscle was seen (Figure 1). 

**Figure 2:**
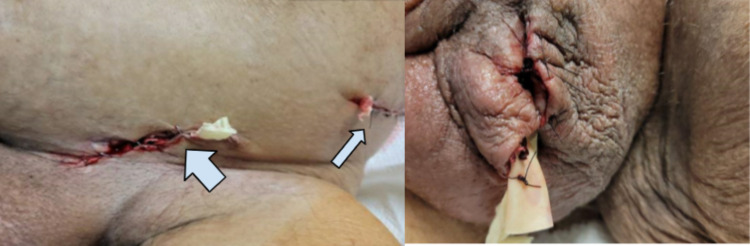
Intraoperative images The left panel of the figure: Left inguinal incision (thick arrow) and left flank incision (thin arrow) The right panel of the figure: Left scrotal incision after changing of packing with Penrose drain (Cardinal Health Inc., Dublin, USA)

The patient was emergently brought to the operating room (OR) for surgical exploration and debridement of the left retroperitoneum, left groin, and scrotum. The left retroperitoneal space was accessed via a flank incision, where there was a significant amount of purulent fluid which was collected for culture. Blunt dissection of the left retroperitoneal space along the psoas muscle up to the lower pole of the kidney was performed. An organized hematoma was discovered in the left retroperitoneum which was removed. Additional incisions were made in the left inguinal region and the left scrotum (Figure 2).

On dissection of the scrotum, there was a significant amount of purulence within the Dartos muscle surrounding the testicle. The tunica vaginalis was also found to be infected. Testicular tissue and the spermatic cord appeared to be gangrenous. The left inguinal incision extended the inguinal canal from the external to the internal inguinal ring. The spermatic cord was then dissected up to the internal inguinal ring due to infection disrupting the fascial plane. Ultimately, an orchiectomy was performed due to the apparent extension of the primary infection from the retroperitoneum. The left proximal spermatic cord stump was sent for tissue culture due to the gangrenous appearance of the spermatic cord structures within the tunica vaginalis. 

On postoperative day one, the WBC count was 9.4 x 10^3^ per uL and lactate levels decreased to 0.5 mg/dL. Scrotal surgical cultures and abdominal cultures revealed *Escherichia Coli*. The left testicle post-orchiectomy tissue exam found acute inflammation and necrosis involving the spermatic cord. The scrotal tissue pathology also revealed acute inflammation and necrosis. On postoperative day three, piperacillin-tazobactam was discontinued. Daily intravenous meropenem and a single dose of gentamicin were initiated secondary to reported sensitivities from the cultures positive for *Escherichia coli* collected during surgery. 

On postoperative day six, packing was removed from the retroperitoneum and revealed some purulent discharge from the site. A 19 French Blake drain (Ethicon, Cincinnati, USA) was digitally inserted into the patient’s left retroperitoneum at the bedside. A Davol drain (Davol Inc., Warwick, USA) was also placed but this drain did not maintain suction. The scrotum was also repacked, after digitally exploring the space, with a canister of iodoform gauze (Covidien, Mansfield, USA). 

The 19 French Blake drain was removed on postoperative day 10. The patient was ultimately discharged on postoperative day 10 with planned daily packing changes. A follow-up CT scan was performed two months postoperatively which did not show any residual drainable collection or evidence of infection. 

## Discussion

**Figure 3:**
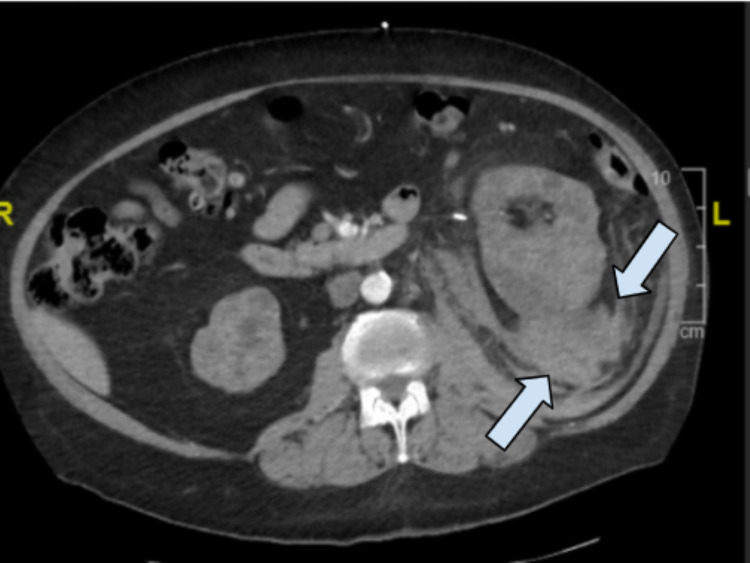
CT transverse imaging seven months prior to presentation Arrows show the 5.8 x 3.9 x 8.7 cm left perinephric hematoma

We report a case of emphysematous pyelonephritis that extended down to the scrotum causing Fournier’s gangrene requiring left orchiectomy in a 61-year-old male. We hypothesize the initial infection arose from a left retroperitoneal perinephric hematoma and tracked retroperitoneally. Seven months prior to presentation, the patient had gross hematuria with a decrease in hemoglobin. CT and ultrasound imaging displayed multiple cysts within the left kidney with potential hemorrhagic conversion. Diagnosis of polycystic kidney disease was suspected due to multiple bilateral cysts with end-stage renal disease, but was never confirmed. A cystoscopy and bilateral retrograde pyelogram were performed for further diagnostic evaluation of the patient’s recurrent gross hematuria. Prior to injection of contrast for the left retrograde pyelogram, blood was noted from the left ureteric orifice. Consequently, a left ureteroscopy was performed at that time. Intraoperatively, the bleeding appeared as if it had been derived from the left kidney parenchyma; however, no active bleeding site was detected, and a left nephroureteral stent was placed which was removed after one-month duration. On computed tomography angiography (CTA) imaging of the abdomen and pelvis seven days postoperatively, a large retroperitoneal hematoma was seen on the left, posterior to the left kidney, as well as a large exophytic heterogeneous structure (5.8 x 3.9 x 8.7 cm) of hyperdense material likely representing blood products (Figure 3). 

This retroperitoneal perinephric hematoma may have developed secondary to the hemorrhagic conversion of an existing renal cyst (previously measured 3.8 x 2.7 x 3.5 cm) and/or iatrogenic-induced via stent placement. The hematoma was managed conservatively and subsequent imaging was done to monitor hematoma progression. Repeat CT imaging of the abdomen and pelvis performed approximately 22 days after initial imaging diagnosis demonstrated an increased size but stable and existing left renal subcapsular and retroperitoneal hematoma. No additional interventions were taken at that time.

It is suspected that this retroperitoneal and perinephric hematoma served as a nidus for primary infection. The gas-forming infection of the kidney then tracked down the retroperitoneum to the scrotum, causing an inferior-tracking Fournier’s gangrene. Although infection did not directly involve the renal parenchyma on imaging, the surrounding structures were involved, thus falling under the more general definition of emphysematous pyelonephritis. Extension of infection from the retroperitoneum to the scrotum has been discussed in previous reports. Colonic perforation with subsequent spread along the retroperitoneal fascial planes resulting in fecal contents in the scrotum has been outlined [15]. Communication of the retroperitoneum and scrotum typically occurs anatomically via dissection through the transversalis fascia through the deep inguinal ring. We suspect our patient’s gangrenous spread occurred via this proposed mechanism. A case report discussed retroperitoneal necrotizing fasciitis that stemmed from Fournier’s gangrene in a patient with HIV [16]. Similar anatomic spread of necrotizing infection was reported; however, this infection started at the scrotum. An additional case of xanthogranulomatous pyelonephritis, a chronic granulomatous process of the renal parenchyma, and emphysematous pyelonephritis complicated by Fournier’s gangrene has been explained in the literature as well [5]. History of chronic nephrolithiasis and obstruction of the renal collecting system was the proposed etiology of the infection in this case report. Fournier’s gangrene has also been documented penetrating the urogenital diaphragm and accessing the inguinal canal via the internal and external fascia of the spermatic cord [17].

Multiple surgical debridements are typically required for the treatment of necrotizing fasciitis, averaging 3.5 debridements per patient [18]. These surgical interventions rarely require debridement of the deep fascia or muscle. Some cases of Fournier’s gangrene report orchiectomy; however, this disease complication is rare because the testes and scrotum do not share fascial planes and blood supply [19]. Fournier’s gangrene more characteristically spreads along the Dartos fascia, Colles’ fascia, and Scarpa’s fascia due to their contiguous layers. This provides a direct route for spread from the abdominal wall to the scrotum [19]. We suspect our patient had a non-classic form of Fournier’s gangrene, whereas the initial retroperitoneal necrotizing infection traversed through the inguinal canal via the spermatic cord to ultimately involve the scrotum and testis. The testicle’s external layer is composed of a fibrous barrier within the tunica albuginea. The external spermatic fascia, cremasteric muscle, internal spermatic fascia, and tunica vaginalis all serve as protective layers in the testes. Infections typically do not traverse these multiple anatomic layers and therefore spare the testes from involvement. Importantly, the scrotum receives arterial supply from the anterior and posterior scrotal arteries whereas the testicle receives its blood supply from the aorta [20]. Distinct vasculature to the scrotum and testes decreases the risk of transmission of bloodborne infection. Cases of testicular involvement in Fournier’s gangrene often suggest retroperitoneal origin or spread of primary infection [15]. In our patient, involvement of the scrotum as well as paratesticular tissue and spermatic cord necessitating orchiectomy displayed the extent of infection beyond local fascial extension as well as retroperitoneal spread.

## Conclusions

This case report describes a rare occurrence of an infected perinephric hematoma complicated by emphysematous pyelonephritis with tracking to the scrotum resulting in Fournier’s gangrene with testicular involvement. We recommend increased surveillance via imaging, including CT abdomen and pelvis, in patients with known renal hematomas to minimize rates of this pathologic transformation.
